# Benign Intracranial Hypertension Due to Hypoparathyroidism: A Case Report

**DOI:** 10.3389/fneur.2021.818638

**Published:** 2022-01-10

**Authors:** Giorgia Sforza, Annalisa Deodati, Romina Moavero, Laura Papetti, Ilaria Frattale, Federico Vigevano, Stefano Cianfarani, Massimiliano Valeriani

**Affiliations:** ^1^Headache Center, Child Neurology Unit, IRCCS Bambino Gesu', Rome, Italy; ^2^Child Endocrinology Unit, Bambino Gesu' Children's Hospital, Rome, Italy; ^3^Child Neurology and Psychiatry Unit, Tor Vergata University of Rome, Rome, Italy; ^4^Dipartimento Pediatrico Universitario Ospedaliero, IRCCS Bambino Gesu' Children's Hospital, Rome, Italy; ^5^Dipartimento di Medicina dei Sistemi, University of Rome Tor Vergata, Rome, Italy; ^6^Department of Women's and Children's Health, Karolinska Institutet and University Hospital, Stockholm, Sweden; ^7^Center for Sensory-Motor Interaction, Aalborg University, Aalborg, Denmark

**Keywords:** headache, idiopathic intracranial hypertension, hypoparathyroidism, children, pseudotumor cerebri (PTC)

## Abstract

**Objective:** The objective of this study is to present the rare case of a young girl with idiopathic intracranial hypertension secondary to hypoparathyroidism.

**Background:** Idiopathic intracranial hypertension is a neurological syndrome characterized by elevated intracranial pressure (> 25 cmH_2_O) in the absence of intracerebral abnormalities or hydrocephalus. The pathophysiology of idiopathic intracranial hypertension is unknown, and rare cases of idiopathic intracranial hypertension secondary to hypoparathyroidism have been described. It is supposed that hypocalcemia causes decrease in the absorption of cerebrospinal fluid in arachnoidal granulations.

**Methods:** The workup of the girl with idiopathic intracranial hypertension and hypoparathyroidism included physical examination, blood tests, diagnostic imaging, and lumbar puncture.

**Results:** We present a 9-year-old female patient who was hospitalized for headache associated with nausea and vomiting for 3 weeks. She underwent an ophthalmologic examination that revealed papilledema. Lumbar puncture revealed an opening pressure of 65 cm H_2_O; cerebrospinal fluid analysis and brain computed tomography scan were normal. The patient started taking acetazolamide. Blood tests revealed hypocalcemia associated with high phosphorus level and undetectable PTH hormone, which led us to suspect hypoparathyroidism. She had never had cramps, paraesthesias, or tetany. Chvostek's and Trousseau's signs were positive. In the neck ultrasonography, parathyroids were not visible. Oral supplementation with calcitriol and calcium was started. Headache, nausea, and vomiting immediately disappeared after the lumbar puncture, and the papilledema improved gradually.

**Conclusions:** Several anecdotal cases of idiopathic intracranial hypertension secondary to hypoparathyroidism have been described. However, our case report is of particular interest, since the child did not present with typical neurological hypoparathyroidism symptoms. Therefore, we recommend that hypoparathyroidism should be included in diagnostic investigations on children with clinical findings of idiopathic intracranial hypertension, because clinical manifestations of hypoparathyroidism are variable and may involve almost all organ systems.

## Introduction

Idiopathic intracranial hypertension (IIH), also known as pseudotumor cerebri (PTC), is a rare disorder characterized by increased intracranial pressure (ICP) in the absence of any brain parenchymal lesions, vascular malformations, hydrocephalus, or central nervous system (CNS) infections with normal cerebrospinal fluid composition and neuroimaging (1, 2). It is characterized by clinical manifestations such as symptoms and signs of intracranial hypertension. Patients can present with papilledema and/or visual loss, but headache is certainly the most common presenting symptom (1). However, headache's features in patients with IIH are widely variable and not specific. Pain is often referred as unusually severe and can be throbbing or pulsatile; it can also be lateralized (3).

Diagnosis in the pediatric population (1–18 years) is confirmed by an opening pressure (OP) of cerebrospinal fluid (CSF) higher than 28 cmH_2_O (while the cutoff in adults is established at 25 cmH_2_O). The estimated annual incidence of IIH in the general population is 1–2 per 100,000 (4), and it is higher in obese women of childbearing age (4–21 per 100,000) (4, 5).

The pathogenesis of IIH is still largely unknown. ICP is determined by balance between production and absorption of cerebrospinal fluid (CSF). According to the Monro-Kellie rule, an increase in ICP might be related to increased CSF, expanded brain tissue, or increased blood volume (6). Therefore, proposed hypotheses include excess in CSF production, CSF outflow reduction, increase in cerebral blood volume and/or brain water content, obstruction to the venous system, endocrinological or metabolic causes, chronic inflammation, and obesity (in pre- and post-pubertal females) (7, 8).

Although typically diagnosed in obese women of childbearing age, IIH can also occur in males, younger children, older adults, and patients who are not overweight (9). In atypical cases, investigations aimed at looking for secondary causes of intracranial hypertension are very important. Beyond space occupying lesions, a number of systemic diseases (adrenal insufficiency, renal failure, systemic lupus erythematosus, and infections), drugs (vitamin A, amiodarone, cyclosporine, tetracyclines, and lithium), vitamin deficiency (vitamin D deficiency) and excess (hypervitaminosis A), and hereditary conditions (trisomy 21, Chiari malformation, and hydrocephalus) can cause intracranial hypertension (10).

In this study, we illustrate the association between idiopathic intracranial hypertension and hypoparathyroidism (HPTH) in a non-overweight 9-year-old child.

## Case Description

A 9-year-old female patient was hospitalized for headache associated with nausea and vomiting that lasted for 3 weeks. Her previous neurological history was unremarkable, with the exception of a diagnosis of attention deficit disorder. Familial history was positive for thyroiditis (mother and grandmother). Her body mass index (BMI) was 20 (normal).

Initially, she underwent an ophthalmologic examination that showed bilateral papilledema. The brain CT scan was normal. Lumbar puncture (LP) revealed an opening pressure of 65 cm H_2_O, with normal CSF chemical composition (Table 1). Therefore, we diagnosed IIH, and the patient started acetazolamide 375 mg/day. However, the blood examination showed hypocalcemia (6.3 mg/dl, normal values: 8.5–10.5) associated with high phosphorus level and undetectable PTH hormone (3 pg/ml), which led us to suspect HPTH and mild defect of vitamin D (26.8 ng/ml; normal value > 30 ng/ml) (Table 2). Pituitary, thyroid, and adrenal function was normal.

**Table 1:** Cerebrospinal fluid (CSF) chemical analysis of the patient at admission to our department.

**CSF examination**	**Values**	**Normal Values**
Color	colorless	-
Feature	clear	-
Protein (mg/dL)	13.9	<45
Glucose (mg/dL)	48	32–82
WBC (/mm3)	1	0–5
RBC(/mm3)	0	0

**Table 2:** Chemical analysis of our patient showing decreased calcium level associated with increased phosphorus and low PTH values, and suggesting hypoparathyroidism (HPTH).

**Blood and urine test**	**Values**	**Normal Values**
Serum calcium	6.3 mg/dL	8.5–10.5
Serum phosphorus	10.2 mg/dL	3.7–5.6
Serum parathormone	3 pg/mL	15–65
Serum albumin	5 g/dL	3.5–5.5
Urine calcium–creatinine ratio	0 mg/mg	0–0.25
Serum vitamin D	26.8 ng/mL	20–100

Neck ultrasonography showed normal thyroid, but the parathyroids were not visible. The patient had never had cramps, paraesthesias, or tetany, but Chvostek's and Trousseau's signs were found. Oral supplementation with calcitriol (0.5 mcg/day) and calcium (500 mg/day) was started. While headache, nausea, and vomiting disappeared immediately after the LP, the papilledema improved gradually with resolution in a period of 2 months from the start of her symptoms. To exclude congenital hypoparathyroidism (DiGeorge syndrome), immunological tests and multiplex ligation-dependent probe amplification (MLPA) analysis of chromosome 22 were carried out and were normal.

## Discussion And Conclusion

Several anecdotal cases of IIH secondary to hypoparathyroidism (HPTH) have been described (11–15). Although pathophysiological mechanisms underlying this association are not known, intracranial hypertension is possibly due to decrease in CSF absorption at the level of arachnoidal granulations, which is restored when calcemia is corrected (1, 11).

Our case report is of particular interest, since the child did not present with typical neurological HPTH symptoms, such as tetany, cramps, paraesthesias, seizures, behavioral disorders, and intracranial calcifications. Only the serum calcium dosage led us to suspect this condition. Therefore, we suggest to include this blood test in diagnostic investigations of children with clinical findings of IIH, above all in the absence of evident risk factors for IIH. This is particularly important, since clinical manifestations of HPTH are variable and may involve almost all organ systems; their severity depends on entity and chronicity of hypocalcemia, but treatment with calcium and active vitamin D is easy. Since HPTH can present only with intracranial hypertension, as in our patient, determination of blood calcium level is mandatory in all cases in which IIH is suspected, in order to identify this treatable condition.

## Data Availability Statement

The original contributions presented in the study are included in the article/supplementary material, further inquiries can be directed to the corresponding author/s.

## Ethics Statement

The studies involving human participants were reviewed and approved by Bambino Gesù Hospital Ethics Committee. Written informed consent to participate in this study was provided by the participants' legal guardian/next of kin.

## Author Contributions

MV: conceptualization and supervision. GS and MV: data curation, formal analysis, and investigation. GS: writing—original draft. AD, RM, LP, IF, FV, SC, and MV: writing—review and editing. All the authors have read and agreed to the published version of the manuscript.

## Conflict of Interest

The authors declare that the research was conducted in the absence of any commercial or financial relationships that could be construed as a potential conflict of interest.

## Publisher's Note

All claims expressed in this article are solely those of the authors and do not necessarily represent those of their affiliated organizations, or those of the publisher, the editors and the reviewers. Any product that may be evaluated in this article, or claim that may be made by its manufacturer, is not guaranteed or endorsed by the publisher.

## Abbreviations

IIH: Idiopathic intracranial hypertension
PTC: pseudotumor cerebri
ICP: increased intracranial pressure
CNS: central nervous system
OP: opening pressure
CSF: cerebrospinal fluid
BMI: body mass index
CT: computed tomography
LP: lumbar puncture
MLPA: multiplex ligation-dependent probe amplification.
